# Extracellular vesicles: emerging therapeutic agents for liver fibrosis

**DOI:** 10.20517/evcna.2025.08

**Published:** 2025-05-07

**Authors:** Giulia Chiabotto, Armina Semnani, Elena Ceccotti, Stefania Bruno

**Affiliations:** ^1^Department of Medical Sciences, University of Torino, Torino 10126, Italy.; ^2^Department of Applied Science and Technology, Politecnico di Torino, Turin 10129, Italy.

**Keywords:** Extracellular vesicles, liver fibrosis, hepatic stellate cells, engineered EVs, drug-delivery system

## Abstract

Liver fibrosis is a progressive condition characterized by excessive scar tissue buildup, leading to impaired liver function and potentially cirrhosis. Despite advancements in treatment strategies, effective anti-fibrotic therapies remain an urgent unmet need. Recent research has identified extracellular vesicles (EVs) as promising therapeutic agents due to their ability to mediate intercellular communication and regulate key fibrotic pathways. This review aims to provide a comprehensive overview of the therapeutic potential of EVs in different *in vitro* and *in vivo* models of hepatic fibrosis, focusing on their natural effects and recent advancements in their engineering for enhanced efficacy. EVs can be derived from various cellular sources, including mesenchymal stromal cells (MSCs) and liver-resident cells. Biological materials, including serum, breast milk, bacteria, and plants, also serve as EV sources. Among these, MSC-EVs stand out for their therapeutic potential, which can be significantly enhanced through preconditioning with inflammatory signals, pharmacological agents, or genetic engineering to improve EV quality and efficacy. Engineering techniques have further expanded EV applications, enabling their use as precise and effective drug-delivery vehicles. Approaches such as loading EVs with pharmacological compounds, designing customized EVs, and creating EV-liposome hybrids enable targeted delivery to activated hepatic stellate cells (HSCs), central drivers of fibrosis progression. These strategies enhance the efficacy of EV-based treatments. Both natural and engineered EVs regulate critical pathways of liver fibrosis development, including activation of HSCs, modulation of pro-fibrotic genes, extracellular matrix deposition, and programmed cell death. Additionally, EVs modulate immune responses, fostering a liver microenvironment conducive to repair and regeneration. Combining the natural regenerative properties of EVs with innovative engineering strategies provides highly targeted, effective treatment approaches to restore liver function and address the urgent unmet need for chronic liver disease therapies.

## INTRODUCTION

Liver fibrosis is a complex pathological condition characterized by the excessive accumulation of extracellular matrix (ECM) proteins, including collagen, within the liver^[1]^. It arises as a wound-healing response to chronic liver injury, where normal tissue architecture is progressively replaced by fibrotic tissue. Liver fibrosis can result from various chronic insults to the liver. The most common causes include chronic viral hepatitis, alcoholic liver disease, metabolic dysfunction-associated steatohepatitis (MASH), cholestatic diseases, autoimmune hepatitis (AIH), and genetic and metabolic disorders. Liver fibrosis disrupts the organ structure and impairs its vital functions, including metabolism, detoxification, bile production, and protein synthesis. Liver fibrosis can lead to significant clinical complications. If left unchecked, fibrosis may progress to cirrhosis, which is characterized by extensive scarring, loss of normal liver architecture, and the development of complications such as portal hypertension, liver failure, and an increased risk of hepatocellular carcinoma (HCC)^[2,3]^.

Liver fibrosis is a dynamic process and, under certain conditions, can be reversed, mainly if the underlying cause of injury is addressed early. This reversibility highlights the importance of timely interventions and the potential therapeutic implications of targeting fibrotic pathways^[4]^. Recent preclinical studies highlighted the importance of cell-free therapies based on specific cell-derived bio-products, the extracellular vesicles (EVs)^[5]^. EVs of different sources showed regenerative properties thanks to their biological cargo, which is involved in the reduction of pro-inflammatory molecule secretion and in the targeting of key players of fibrosis, such as activated hepatic stellate cells (HSCs). For these reasons, EVs represent an innovative and promising option to counteract the progression of liver fibrosis by ameliorating organ morphology and function.

## EVs

EVs are membrane-bound particles naturally released from cells^[6]^. EVs consist of aqueous compartments containing diverse biomolecules from the parent cell, including lipids, proteins, nucleic acids, and soluble small molecules^[7,8]^. EVs are considered key mediators of intercellular communication, influencing a wide range of physiological and pathological processes, and have potential applications in diagnostics and therapeutics. EVs can be classified according to the mode of release. The large membrane vesicles (100-1,000 nm), also called microvesicles (MVs), are secreted by shedding or budding from the plasma membrane. Moreover, exosomes (50-100 nm) originate from internal cell compartments and are released via multivesicular bodies (MVBs), while ectosomes (50-200 nm) are released from the cell surface. Lastly, the apoptotic bodies (50-500 nm) are released from the plasma membrane blebbing of apoptotic cells^[5,9,10]^.

EVs can be found in various biological fluids, including blood, urine, saliva, cerebrospinal fluid, and breast milk. EVs released from cells in different tissues and biofluids provide a rich tapestry of information about the diversity of tissues and cellular sources^[11,12]^. Key sources include immune cells that release EVs that modulate immune responses, and stem cells that release EVs that contribute to tissue regeneration and repair^[13]^. EVs produced by adult stem cells, particularly mesenchymal stem cells (MSCs), have critical applications in many sectors, showcasing their versatility. Before, it was thought that MSCs exclusively secreted molecules such as cytokines, chemokines, and growth factors^[14]^. Nevertheless, research has demonstrated that MSCs can also release EVs reacting to chemical, mechanical, and environmental stimuli^[15]^. These MSC-derived EVs (MSC-EVs) expressed specific molecules, including CD9, CD90, CD29, CD73, and CD44. MSC-EVs, with their extensive research and significant role as regeneration drivers, have sparked interest in regenerative medicine. The therapeutic effects of MSCs, coupled with the innovative potential of their secretory factors, particularly EVs, could revolutionize therapeutic approaches.

## PATHOGENESIS OF LIVER FIBROSIS

**Figure 1:**
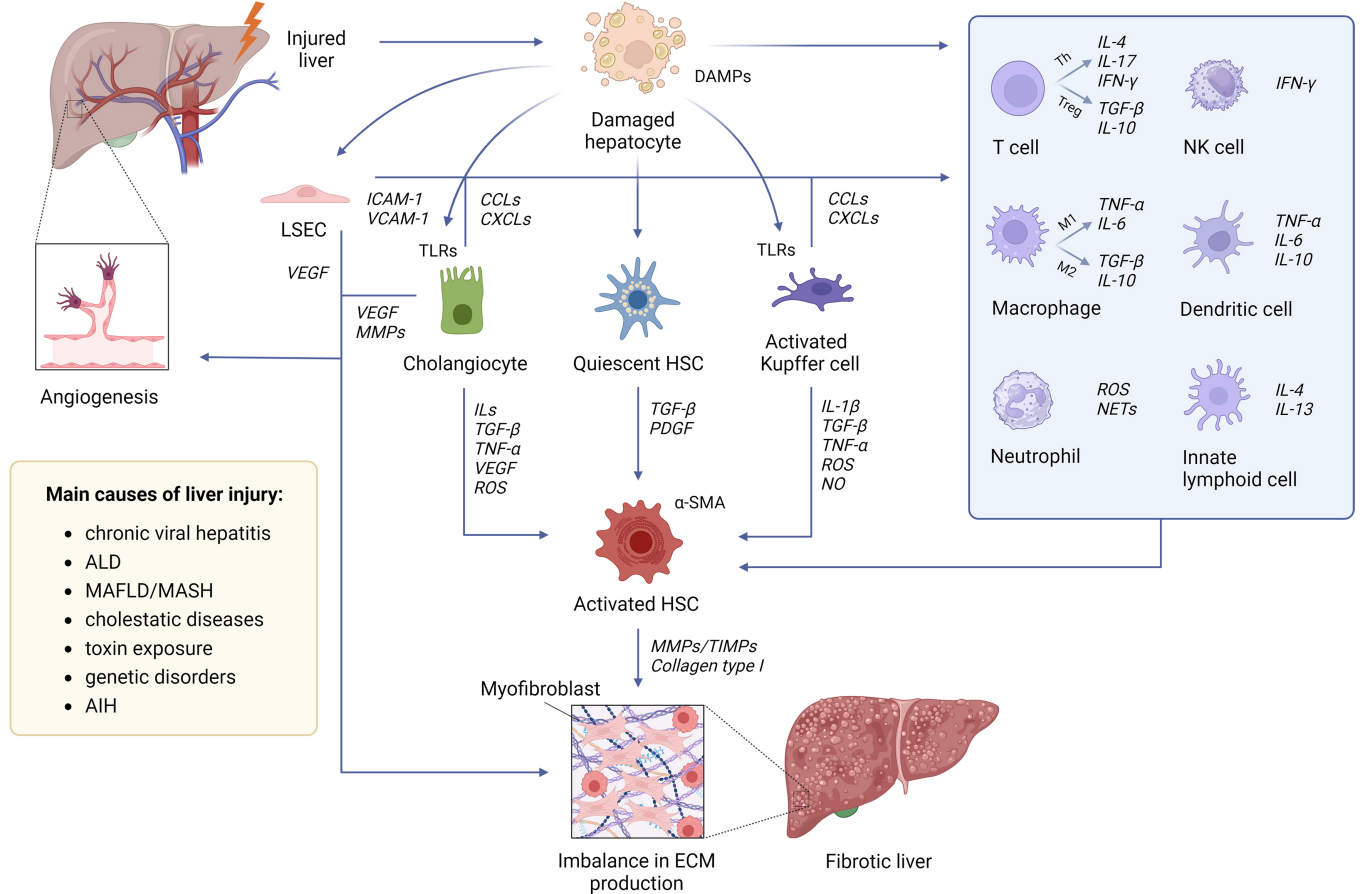
Cells involved in the onset of hepatic fibrosis. Liver fibrosis involves the interplay of several liver-resident and immune cell types. In response to liver injury from various causes, damaged hepatocytes trigger an inflammatory cascade, activating macrophages and promoting the release of ROS and pro-inflammatory cytokines, like TGF-β1. These signals stimulate the activation of quiescent HSCs into myofibroblasts. Activated HSCs proliferate in response to cytokines such as TGF-β and PDGF, producing type I collagen and extracellular matrix components, which drive fibrotic tissue deposition and scar formation. Created using BioRender. Chiabotto, G. (2025) https://BioRender.com/pbjeea3. AIH: Autoimmune hepatitis; ALD: alcohol-associated liver disease; CCLs: CC chemokine ligands; CXCL: CXC chemokine ligand; ECM: extracellular matrix; DAMPs: damage-associated molecular patterns; IFN-γ: interferon-γ; ILs: interleukins; LSEC: liver sinusoid endothelial cell; MAFLD: metabolic-associated fatty liver disease; MASH: metabolic dysfunction-associated steatohepatitis; MMPs: matrix metalloproteinases; NETs: neutrophil extracellular traps; NK: natural killer; NO: nitric oxide; PDGF: platelet-derived growth factor; ROS: reactive oxygen species; TGF-β: transforming growth factor-β; Th: T helper; TNF-α: tumor necrosis factor-α; Treg: T regulatory; VEGF: vascular endothelial growth factor; α-SMA: alpha-smooth muscle actin; TIMPs: tissue inhibitors of metalloproteinases; TLRs: Toll-like receptors; TGF-β1: transforming growth factor-β1; HSCs: hepatic stellate cells.

The pathogenesis of liver fibrosis involves a complex interplay of cellular and molecular mechanisms [Figure 1]. The initiation phase of liver fibrosis is primarily triggered by hepatocyte injury and the subsequent inflammatory response^[16]^. Damaged hepatocytes release damage-associated molecular patterns (DAMPs), which are intracellular molecules that act as “danger signals” and initiate a cascade of molecular and cellular events that activate the fibrotic response. Inflammation is a cornerstone of the initiation phase. The inflammatory process begins with the activation of resident immune cells and the recruitment of circulating immune cells. Kupffer cells, the liver resident macrophages, are among the first responders to hepatocyte injury. They recognize DAMPs and pathogen-associated molecular patterns (PAMPs), leading to release of pro-inflammatory cytokines [e.g., tumor necrosis factor-α (TNF-α), interleukin-1β (IL-1β), interleukin-6 (IL-6)], production of chemokines (e.g., CCL2, CCL5), which recruit monocytes, neutrophils, and lymphocytes to the site of injury, and generation of reactive oxygen species (ROS) and nitric oxide, further amplifying oxidative stress^[17]^. The inflammatory signals attract circulating immune cells, including monocytes, which differentiate into macrophages that perpetuate inflammation and release fibrogenic signals. Neutrophils contribute to tissue damage through degranulation and ROS production; activated T cells release cytokines such as interferon-γ (IFN-γ), driving inflammation and hepatocyte apoptosis. Platelets accumulate at sites of liver injury and release pro-inflammatory mediators, such as serotonin and platelet-derived growth factor (PDGF), which contribute to inflammation^[18]^.

Central to the process of fibrosis development is the persistent activation of HSCs, the liver’s primary fibrogenic cells^[16]^. The activation of HSCs is a pivotal event in developing liver fibrosis. HSCs reside in the space of Disse between hepatocytes and sinusoidal endothelial cells and are typically quiescent in a healthy liver. In this state, they serve as storage cells for vitamin A and other retinoids. Upon liver injury, however, HSCs undergo a dramatic transformation into proliferative, migratory, and fibrogenic myofibroblast-like cells, which are the primary producers of ECM components, including collagen^[19]^.

HSC activation results from dynamic crosstalk between various liver cells [Figure 1]. Injured hepatocytes release DAMPs, ROS, and apoptotic bodies, activating signals for HSCs. Kupffer cells secrete pro-inflammatory cytokines (e.g., TNF-α, IL-1β) and transforming growth factor (TGF)-β, directly activating HSCs. Infiltrating immune cells, including monocytes, neutrophils, and T cells, release cytokines and ROS that influence HSC activation^[20,21]^.

ECM remodeling is a critical process in the pathogenesis of liver fibrosis. In healthy livers, ECM components are produced and degraded in a balanced manner. The liver sinusoidal endothelial cells (LSECs), hepatocytes, Kupffer cells, and HSCs all participate in ECM homeostasis through the secretion and degradation of ECM proteins. In liver fibrosis, the normal process of ECM turnover becomes disrupted. The balance between ECM deposition and degradation is skewed toward excessive ECM production and insufficient degradation, leading to fibrosis progression^[22,23]^. In healthy tissue, matrix metalloproteinases (MMPs), including MMP-2 and MMP-9, degrade collagen and other ECM components, allowing for tissue remodeling and repair. However, in liver fibrosis, the activity of MMPs is inhibited by the overexpression of their inhibitors, leading to a decrease in ECM degradation. The imbalance between MMPs and tissue inhibitors of metalloproteinases (TIMPs) results in accumulating insoluble collagen fibers and other ECM proteins, contributing to fibrosis progression^[24]^.

## THERAPEUTIC BENEFITS OF MSC-EVs IN LIVER FIBROSIS

**Figure 2:**
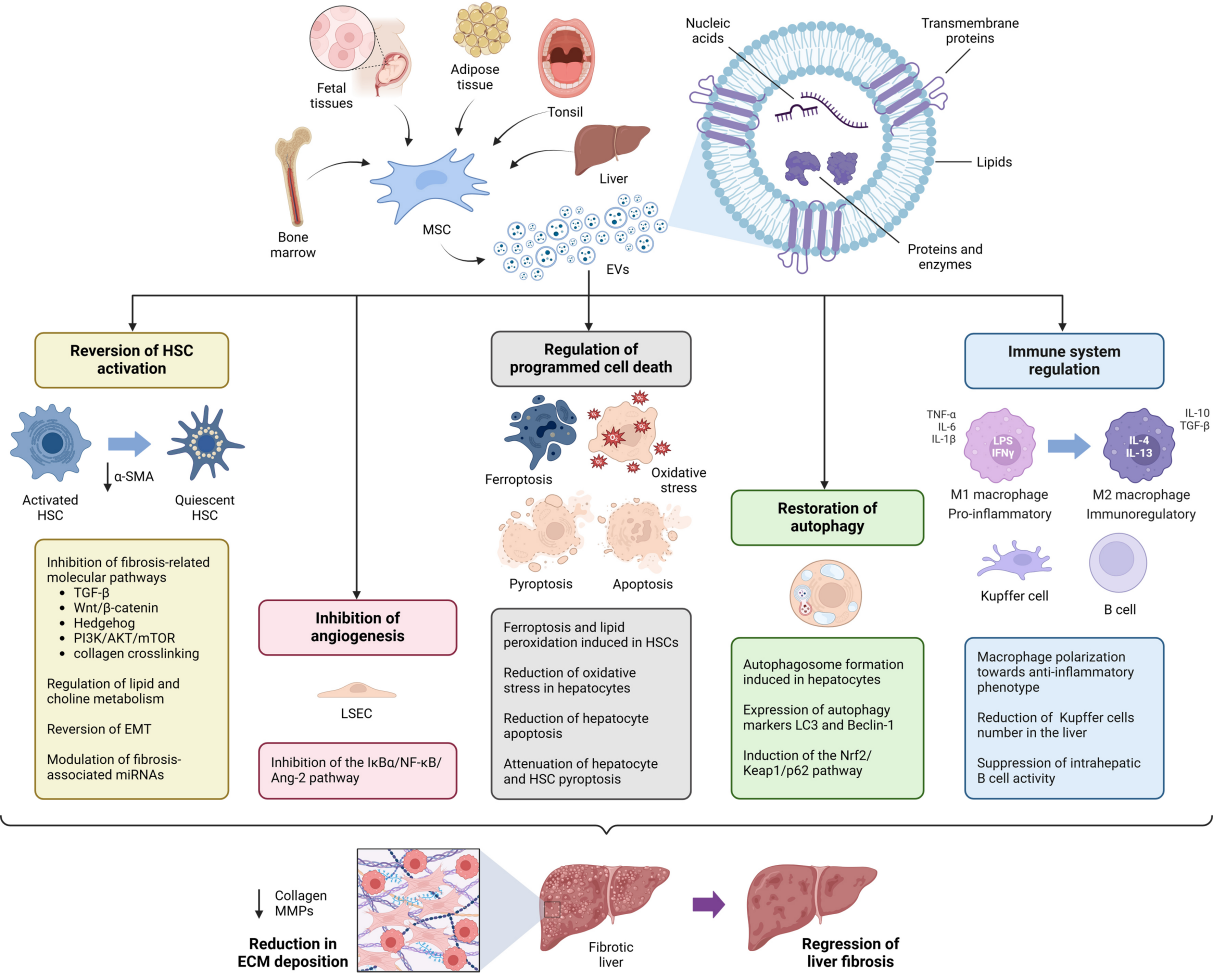
Therapeutic effects of MSC-EVs in liver fibrosis. MSC-EVs from various sources exert multiple therapeutic effects in liver fibrosis by delivering bioactive molecules, including proteins, lipids, and nucleic acids, to liver cells. Key mechanisms include the reversion of HSC activation, mainly through the inhibition of fibrosis-related pathways. MSC-EVs also regulate apoptosis by inducing ferroptosis and lipid peroxidation in HSCs while reducing oxidative stress, necroptosis, and pyroptosis in hepatocytes. Additionally, they promote autophagy, suppress angiogenesis, and modulate immune responses, particularly by driving macrophage polarization toward an anti-inflammatory M2 phenotype. Collectively, these effects contribute to fibrosis attenuation and liver tissue regeneration. Created using BioRender. Chiabotto, G. (2025) https://BioRender.com/aitz8fh. Ang-2: Angiopoietin-2; Beclin-1: autophagy-related protein beclin-1; EMT: epithelial-mesenchymal transition; EVs: extracellular vesicles; HSC: hepatic stellate cell; IFN-γ: interferon-gamma; IkBα: inhibitor of nuclear factor kappa b alpha; IL-4: interleukin-4; IL-6: interleukin-6; IL-10: interleukin-10; IL-13: interleukin-13; Keap1: Kelch-like ECH-associated protein 1; LC3: microtubule-associated protein 1 light chain 3; LPS: lipopolysaccharide; MSC-EVs: mesenchymal stromal cell-derived extracellular vesicles; NF-κB: nuclear factor kappa B; Nrf2: nuclear factor erythroid 2-related factor 2; PI3K/AKT/mTOR: phosphoinositide 3-kinase/protein kinase b/mammalian target of rapamycin; TGF-β: transforming growth factor-beta; TNF-α: tumor necrosis factor-alpha.

MSC-EVs can mitigate liver fibrosis in different *in vitro* and *in vivo* models by influencing crucial mechanisms involved in fibrosis development, such as the activation of HSCs, the regulation of the programmed cell death and the immune cell responses [Figure 2 and Table 1].

**Table 1:** Effects of MSC-EVs isolated from different tissues in liver fibrosis

**MSC-EVs source**	**Therapeutic effects and mechanisms of action**	**Liver fibrosis models**	**References**
Wharton’s Jelly	Inhibition of TGF-β signaling and collagen deposition	TGF-β/cholesterol-treated LX-2	[25-27]
	Monocyte reprogramming into M2-like anti-inflammatory macrophages and T cell inhibition	Macrophages, LX-2, T cells	[28]
Umbilical cord	Inhibition of TGF-β and Hedgehog signaling, reduction of collagen deposition and pathological angiogenesis, reversion of EMT	TGF-β-treated LX-2 and liver spheroids, CCl_4_, TAA, MASH	[29-38]
	Ferroptosis induction	CCl_4_	[39]
	Macrophage polarization toward M2 phenotype	Macrophages, CCl_4_, MCD diet	[40,41]
	Reduction of liver granuloma, inflammation and fibrosis	S. japonicum	[34]
Placenta	Reduction of collagen deposition	TGF-β-treated LX-2 and liver organoids	[42]
Amnion	Inhibition of TGF-β signaling and collagen deposition	LX-2, CCl_4_	[43-45]
	Macrophage polarization toward M2 phenotype	LPS-treated Kupffer cells, macrophages, CCl_4_, MCD diet	[43,44]
Embryo	Inhibition of TGF-β signaling	TGF-β-treated LX-2, CCl_4_	[46]
Bone marrow	Inhibition of TGF-β, Hedgehog and Wnt/β-catenin signaling, reduction of collagen deposition	TGF-β-treated LX-2 and liver spheroids, CCl_4_, DEN	[38,47-52]
	Autophagy restoration	D-GalN/LPS-treated hepatocytes	[53]
	Inhibition of pyroptosis	CCl_4_-induced hepatocytes, CCl_4_	[54]
	Suppression of intrahepatic B cell activity	B cells, CCl_4_, MCD diet	[55]
	Reduction of liver granuloma, inflammation and fibrosis	S. japonicum	[56]
Adipose tissue	Inhibition of TGF-β signaling, reduction of collagen deposition and pathological angiogenesis, reversion of EMT	TGF-β-treated LX-2, CCl_4_, TAA, DEN	[57-62]
	Autophagy restoration via Nrf2/Keap1/p62 pathway	CCl_4_	[59]
	Macrophage polarization toward M2 phenotype	Macrophages, CCl_4_, WD/LPS	[63,64]
Tonsil	Inhibition of Hedgehog signaling and collagen deposition	TGF-β-treated LX-2, CCl_4_	[65]
Liver	Inhibition of TGF-β signaling and collagen deposition	TGF-β-treated LX-2, CCl_4_, MCD diet	[66-68]
	Macrophage polarization toward M2 phenotype	Macrophages, CCl_4_	[69]

CCl4: Carbon tetrachloride; DEN: diethylnitrosamine; EMT: epithelial-to-mesenchymal transition; LPS: lipopolysaccharides; MASH: metabolic dysfunction-associated steatohepatitis; MCD: methionine-choline-deficient; TGF-β: transforming growth factor-beta; WD: western diet.

### MSC-EVs regulate HSC activation and modulate pro-fibrotic pathways

Recent findings highlight the potential of MSC-EVs to effectively inhibit TGF-β expression and suppress HSC activation. TGF-β is crucial for wound healing, tissue homeostasis, and embryonic development, with functions extending to cell proliferation, senescence, apoptosis, inflammatory responses, tissue fibrosis, and aging. Among the numerous isoforms of TGF-β, TGF-β1 is the most relevant to hepatic fibrosis, as it promotes the activation of HSCs via the TGF-β1/Smads signaling pathway^[70,71]^.

Fetal tissue-derived MSC-EVs, particularly those from the umbilical cord and Wharton’s jelly, known for their abundance of MSCs, have shown significant promise in the treatment of hepatic fibrosis^[72]^. EVs isolated from Wharton’s jelly MSCs (WJ-MSC-EVs) can inhibit HSC activation by partially restoring the expression of miR-133a, whose downregulation is associated with fibrosis progression^[25]^. *In vitro*, WJ-MSC-EVs substantially reduced the expression of *NOX1*, *NOX2*, and *NOX4* genes, which are closely associated with TGF-β signaling, as well as the TGF-β/Smad3 pathway, resulting in a decrease of ECM accumulation^[25,26]^. Moreover, exosomes isolated from WJ-MSCs have been shown to reduce cholesterol-induced liver fibrosis by inhibiting the phosphorylation of the Smad3 protein in LX-2, a HSC human cell line^[27]^.

EVs derived from umbilical cord MSCs (UC-MSC-EVs) demonstrated significant anti-fibrotic potential^[29-38]^. In a thioacetamide (TAA)-induced liver fibrosis model, UC-MSCs-EVs exerted therapeutic effects through the inhibition of the Hedgehog/Smoothened signaling pathway, which plays a key role in HSC activation^[29]^. Notably, UC-MSC-EVs reversed the expression of epithelial-to-mesenchymal transition (EMT) markers induced by TGF-β in HSCs, both *in vitro* and *in vivo*, as shown in a carbon tetrachloride (CCl_4_)-induced liver fibrosis model^[30]^. On a molecular level, these EVs inhibited HSC activation by modulating the miR-148a-5p/SLIT3 axis, providing deeper insights into their anti-fibrotic mechanism of action^[31]^. By interfering with enzymes like lysyl oxidase (LOX) that mediate collagen crosslinking, UC-MSC-EVs contribute to maintaining ECM flexibility and reduce fibrosis progression^[32]^. In a MASH model induced by a methionine-choline-deficient (MCD) diet, UC-MSC-EVs attenuated liver fibrosis by inhibiting LSEC angiogenesis via USP9X-mediated modulation of the IκBα/NF-κB/Ang-2 pathway^[33]^. Comparably to UC-MSC-EVs, EVs from placenta-derived MSCs suppressed collagen I expression via the miR-378c/SKP2 axis, effectively downregulating ECM components in liver fibrosis 2D and 3D *in vitro* models^[42]^. Amnion-derived MSC-EVs (AMSC-EVs) downregulated TGF-β1 signaling, reducing HSC activation and collagen production^[43,44]^. Additionally, they delivered miR-200a to hepatocytes, inhibiting PIK3R3 transcription via suppression of ZEB, thereby mitigating hepatic fibrosis^[45]^. Likewise, miR-6766-3p-enriched exosomes from human embryonic stem cells (ESCs) have been shown to significantly reduce the expression of pro-fibrotic markers in LX-2 cells. This effect is achieved through the inhibition of TGFβRII and Smad signaling, leading to decreased HSC activation and the prevention of hepatic fibrosis^[46]^.

EVs derived from human bone marrow MSCs (BMSC-EVs) exhibit potent anti-fibrotic properties comparable to those observed with fetal MSC-EVs. *In vitro*, both BMSC-EVs and UC-MSC-EVs effectively suppressed the expression of pro-fibrotic markers in TGF-β1-activated liver spheroids and HSCs^[38]^. In a CCl_4_-induced liver fibrosis model, the administration of BMSC-EVs significantly alleviated liver fibrosis by reducing collagen deposition, improving liver functionality, suppressing inflammation, and promoting hepatocyte regeneration^[47,48]^. Mechanistically, BMSC-EVs inhibited the expression of key components of the Wnt/β-catenin signaling pathway, such as PPARγ, Wnt3a, Wnt10b, β-catenin, WISP1, and Cyclin D1, alongside fibrogenic markers like alpha-smooth muscle actin (α-SMA) and collagen I, in both activated HSCs and fibrotic liver tissues^[47]^. Additional anti-fibrotic mechanisms include BMSC-EV-derived miR-618 targeting Smad4, a key mediator in the TGF-β/Smad pathway^[49]^, and circCDK13 regulating MFGE8 through the miR-17-5p/KAT2B axis^[50]^. Specifically, MFGE8 strongly inhibited TGF-β1-induced HSC activation and mitigated liver fibrosis^[51]^. In a diethylnitrosamine-induced liver fibrosis model, BMSC-EVs combined with the antihistamine drug rupatadine demonstrated significant anti-fibrotic efficacy by attenuating oxidative stress, inflammation, and necroptosis. This combination therapy upregulated miR-200a levels, leading to reduced expression of key fibrotic markers, including TGF-β1 and α-SMA, while inhibiting the Hedgehog signaling pathway, a critical driver of HSC activation^[52]^.

In a TAA-induced liver fibrosis model, EVs isolated from adipose tissue-derived MSCs (ADSCs-EVs) significantly suppressed the expression of fibrogenic markers, including MMP-2, collagen-1, and α-SMA, while accumulating in fibrotic liver tissue to restore liver functionality^[57]^. In a CCl_4_-induced liver fibrosis model, ADSC-EVs alleviated hepatic fibrosis by inhibiting HSC activation, and reducing collagen deposition, EMT, and liver inflammation^[58,59]^. Mechanistically, ADSC-EVs exploited their anti-fibrotic activity by targeting the PI3K/AKT/mTOR signaling pathway and regulating lipid and choline metabolism, with CHPT1 playing a role in vesicular membrane maintenance^[58]^. Additionally, ADSC-EVs have been shown to remodel glutamine and ammonia metabolism mediated by hepatocellular glutamine synthetase^[60]^. Specific microRNAs (miRNAs) in ADSC-EVs, including miR-150-5p, attenuated fibrosis by inhibiting CXCL1 expression^[61]^, while miR-20a-5p targeted the p38 MAPK/NF-κB pathway via TGFBR2 to mitigate fibrogenesis^[62]^.

Tonsil-derived MSCs (T-MSCs) showed comparable characteristics to BMSCs and ADSCs, including similar morphology, surface marker expression, and robust potential for proliferation and differentiation. EVs derived from T-MSCs (T-MSC-EVs) effectively inhibited HSC activation and mitigated fibrosis in a CCl_4_-induced chronic liver injury model. Notably, T-MSC-EVs are enriched with miR-486-5p, which targets Smoothened to suppress Hedgehog signaling, a key pathway in HSC activation and fibrosis progression^[65]^.

EVs derived from human liver stem cells (HLSC-EVs), an MSC-like population derived from healthy human livers, exhibit potent anti-fibrotic and anti-inflammatory effects. In a murine model of liver fibrosis induced by MCD diet, HLSC-EVs promoted tissue regeneration by delivering bioactive molecules that suppressed fibrogenic pathways and enhanced hepatocyte repair^[66]^. They also influenced the expression of long non-coding RNAs in the liver, pivotal regulators of hepatic fibrosis and inflammation^[67]^. Additionally, *in vitro* studies demonstrated that HLSC-EVs reduce fibrotic markers by modulating TGF-β signaling and inhibiting HSC activation, with their anti-fibrotic effects partially mediated by the delivery of miRNAs, such as miR-146a-5p^[68]^.

### MSC-EVs regulate programmed cell death

In addition to the inhibition of HSC activation, MSC-EVs can influence hepatocyte programmed cell death and proliferation - other important aspects to consider in liver fibrosis^[73]^. MSCs-EVs suppress acetaminophen (APAP)- and H_2_O_2_-induced apoptosis of hepatocytes *in vitro* by positively regulating Bcl-xl expression, and stimulate hepatocyte proliferation *in vivo* in CCL4-induced liver damage^[74]^. Another study shows that MSC-EVs reduce apoptosis by increasing autophagy in hepatocytes. Autophagy is a crucial cellular mechanism responsible for maintaining internal homeostasis by degrading and recycling organelles, proteins, and other macromolecules^[75]^. This process enables cells to adapt to metabolic stress by reusing intracellular components^[76]^. In the liver, autophagy plays dual roles: it can suppress hepatocyte death and regulate macrophage activity, mitigating fibrosis, while also supporting HSC activation, which promotes fibrosis^[77]^. Autophagy is essential for sustaining intrahepatic equilibrium in both parenchymal and non-parenchymal cells, emphasizing its significance in energy balance and the removal of damaged cellular components^[78]^. BMSC-EVs have been shown to enhance autophagy markers such as LC3 and Beclin-1, increasing autophagosome formation in hepatocytes and reducing apoptosis induced by D-galactosamine and lipopolysaccharides (LPS)^[53]^. ADSC-EVs restored autophagy and Nrf2 expression in a CCl_4_-induced liver fibrosis model via Nrf2/Keap1/p62 pathway^[59]^. Thus, leveraging MSC-EVs to regulate autophagy represents a promising therapeutic approach for hepatic fibrosis.

Pyroptosis is another form of programmed cell death associated with inflammation and characterized by gasdermin-mediated cell membrane pore formation, cytokine release, and cellular lysis. It plays a significant role in the progression of liver fibrosis by amplifying inflammatory responses and promoting hepatocyte and HSC damage. In a cirrhosis model, Zhang *et al*. demonstrated that BMSC-EVs inhibited pyroptosis by downregulating the expression of key pyroptotic markers such as gasdermin D (GSDMD), caspase-1, and IL-1β. By mitigating pyroptosis in hepatocytes and HSCs, BMSC-EVs not only reduced inflammation but also ameliorated fibrosis progression, further supporting their role in promoting hepatic regeneration and maintaining tissue integrity^[54]^.

Ferroptosis is a regulated cell death caused by iron-dependent lipid peroxidation, different from apoptosis, cell necrosis, and autophagy. Recent evidence showed that MSC-EVs can induce ferroptosis in HSCs, thereby alleviating liver fibrosis^[39]^. Within UC-MSC-EVs, Tan *et al*. identified BECN1 (Beclin 1), a key regulator of various cellular processes, including autophagy and apoptosis^[39]^. In a CCl_4_-induced liver fibrosis model, BECN1-containing exosomes promoted ferroptosis in HSCs by modulating the cystine/glutamate exchange transporter (xCT) and the glutathione peroxidase 4 (GPX4) enzyme. The xCT antiporter system is critical in maintaining redox balance, while GPX4 protects against oxidative stress and lipid peroxidation. By regulating this axis, UC-MSC-EVs facilitate lipid peroxidation and ferroptosis in HSCs, offering a novel therapeutic strategy for liver fibrosis.

### MSC-EVs regulate macrophage polarization and influence immune cells

Recent studies highlight the contribution of hepatic macrophages in liver fibrosis, where they initiate, perpetuate, and amplify the inflammatory cascade while critically modulating HSC activation^[79]^. Macrophages, which exhibit notable plasticity, are categorized into two primary subpopulations: classically activated (M1) macrophages and alternatively activated (M2) macrophages^[80]^ [Figure 1]. M1 macrophages, typically induced by LPS and IFN-γ, are pro-inflammatory cells able to secret cytokines such as IL-1β, TNF-α, and inducible nitric oxide synthase that contribute to fibrotic processes. Conversely, M2 macrophages, activated by interleukins like IL-4 and IL-13, display anti-inflammatory characteristics, producing factors such as IL-10 and TGF-β, and aid in tissue repair by mitigating inflammation^[81]^.

In a CCl_4_-induced liver fibrosis model, ADSC-EVs localized primarily in liver lesions, with macrophages showing the highest EV uptake^[63]^. In a melanocortin type-4 receptor knockout mouse model fed with a western diet (WD) and injected with LPS, ADSC-EVs administration increased the frequency of anti-inflammatory macrophages in the liver, thus contributing to improved liver function and attenuated inflammation and fibrosis^[64]^.

MSC-EVs have emerged as pivotal regulators of macrophage polarization, effectively shifting M1 macrophages to the anti-inflammatory M2 phenotype^[82]^. This polarization plays a critical role in alleviating hepatic fibrosis. For instance, miR-148a, enriched in UC-MSC-EVs, targets the KLF6 pathway, which is involved in the modulation of macrophage behavior through the JAK/STAT signaling pathway^[40]^. The intravenous administration of UC-MSC-EVs in an MCD-induced liver fibrosis model alleviated hepatic fibrosis by promoting macrophage polarization toward an anti-inflammatory phenotype, leading to increased production of anti-inflammatory cytokines and suppression of pro-inflammatory mediators^[41]^. Similarly, AMSC-EVs reduced hepatic macrophage infiltration, and favored their polarization to a pro-reparative M2-like phenotype both *in vitro* and *in vivo*^[43,44]^. Furthermore, a specific subset of WJ-MSC-EVs has shown potential in reprogramming monocytes into M2-like functional macrophages with enhanced phagocytic activity, which effectively inhibited the activation of LX-2 cells^[28]^. By delivering miR-142-5p, liver stem cell-derived EVs regulated macrophage polarization by targeting cathepsin B expression, thereby reducing the pro-inflammatory M1 phenotype and promoting an anti-inflammatory M2 phenotype^[69]^. This modulation mitigated the inflammatory cascade and fibrosis progression. Based on this evidence, targeting macrophage polarization and leveraging their anti-inflammatory capabilities through MSC-EVs holds great potential for preventing hepatic fibrosis progression.

While the roles of macrophages and Kupffer cells in liver fibrosis are well-established, recent findings by Feng *et al*. revealed that B cells are also critical mediators of liver fibrosis progression^[55]^. In both CCl_4_-induced and MCD diet-induced liver fibrosis models, BMSC-EVs suppressed intrahepatic B cell activity by modulating the MAPK and NF-κB signaling pathways, reducing inflammation and fibrogenesis. These insights provide a novel perspective on the interaction between MSC-EVs and intrahepatic immune cells, expanding their potential applications in the treatment of liver fibrosis.

In addition to toxin-induced liver fibrosis, parasitic infections such as schistosomiasis are major contributors to hepatic fibrogenesis, especially in endemic regions. *Schistosoma* infection drives liver fibrosis through chronic inflammation and granuloma formation around deposited parasite eggs, perpetuated by macrophage activation and T helper type 2-skewed immune responses. With their proven anti-inflammatory and anti-fibrotic properties, both BMSC-EVs^[56]^ and UC-MSCs^[34]^ have shown effectiveness in treating schistosomiasis-induced liver fibrosis, as demonstrated by the improved liver granuloma and the decreased collagen deposition in the livers of schistosome-infected mice.

## THERAPEUTIC EFFECTS OF EVs FROM OTHER SOURCES IN LIVER FIBROSIS

### EVs derived from non-MSC animal sources

Differentiated resident liver cell-derived EVs exhibit anti-fibrotic effects comparable to those of MSC-EVs [Table 2]. EVs from LSECs effectively inhibited HSC activation *in vitro*^[83]^. Primary human and mouse hepatocyte-derived EVs not only suppressed HSC activation *in vitro* but also bound to HSCs *in vivo*, mitigating fibrogenesis, reducing hepatocyte injury, and attenuating inflammation^[84,85]^. Interestingly, chemical-induced hepatocytes (iHep), hepatocyte-like cells derived from ADSCs^[96]^ and from mouse embryonic fibroblasts^[97]^, offer a scalable and sustainable source of EVs due to their capacity for expansion *in vitro*. In a CCl_4_-induced liver fibrosis model, human iHep-EVs suppressed the expression of inflammatory genes and cytokines while inhibiting HSC activation by targeting the TGF-β1/Smad signaling pathway. Moreover, by activating the Nrf2/HO-1 signaling pathway, iHep-EVs reduced oxidative stress, inflammation, and fibrosis^[86]^. Another study investigated the therapeutic effects of iHep-derived apoptotic vesicles (apoVs) released upon stimulation with pro-apoptotic drugs (e.g., staurosporine). *In vitro*, these iHep-apoVs suppressed HSC activation, promoted hepatocyte viability, and induced the conversion of macrophages from an M1 to an M2 phenotype. In an *in vivo* model of CCl_4_-induced liver fibrosis, iHep-apoVs were internalized by both myofibroblasts and macrophages, leading to reduced hepatic fibrosis and inflammation^[87]^.

**Table 2:** Effects of EVs from different sources on liver fibrosis

**EV source**	**Therapeutic effects and mechanisms of action**	**Liver fibrosis models**	**References**
LSEC	Increase of inflammatory gene expression in activated HSCs	Primary rat liver cells	[83]
	Decrease of the inflammatory gene expression in Kupffer cells		
Primary human and mouse hepatocyte	Suppression of fibrogenic gene expression, reduction of inflammation, and protection of hepatocytes from damage	CCl_4_	[84,85]
iHep	Suppression of the expression of inflammatory genes and cytokines	CCl_4_	[86,87]
	Inhibition of HSC activation by targeting the TGF-β1/Smad signaling pathway		
	Activation of the Nrf2/HO-1 signaling pathway and reduction of oxidative stress, inflammation and fibrosis		
iPSC	Reduction of HSC activation and pro-fibrotic markers	Activated human HSC, CCl_4_, BDL	[88]
	Inhibition of HSC proliferation and migration		
	Downregulation of fibrosis-related genes		
	Reduction of collagen deposition and HSC activation *in vivo*		
NK cells	Suppression of TGF-β1-induced HSC proliferation	CCl_4_	[89]
	Reduction of fibrosis		
	Inhibition of HSC activation		
Serum	Reduction of hepatocyte death, inflammation, and fibrosis-associated gene expression, increase of hepatocyte proliferation	CCl_4_, TAA	[90]
	Attenuation of activation of HSCs and reduction of the expression of fibrosis markers	Activated human HSCs	
PRP	Reduction of liver fibrosis	CCl_4_	[91]
	Decrease of serum ALT level		
	Promotion of hepatocyte proliferation		
	Induction of macrophage polarization to an anti-inflammatory phenotype		
Milk	Inhibition of HSC proliferation and collagen expression	Activated HSCs	[92]
	Increase PPAR-γ expression		
Tea leaf	Inhibition of HSC activation	LX-2, CCl_4_	[93]
	Reduction of collagen deposition		
	Decrease of serum AST & ALT levels		
Cannabis bud (Hemp sprout)	Inhibition of HSC activation	LX-2, NAFLD	[94]
	Reduction of collagen deposition		
	Attenuation of liver fibrosis		
Akkermansia muciniphila	Inhibition of HSC activation	LX-2, HFD/CCl_4_	[95]
	Reduction of fibrosis markers		
	Modulation of inflammatory response		
	Improvement of liver and colon histopathology		
	Amelioration of intestinal permeability		

ADSC: Adipose-derived stem cell; ALT: alanine aminotransferase; α-SMA: alpha-smooth muscle actin; AST: aspartate aminotransferase; BDL: bile duct ligation; CCL4: carbon tetrachloride; CCN2: cellular communication network 2; HFD: high-fat diet; HiHep: human induced hepatocyte; HO-1: hemoxygenase 1; HSC: hepatic stellate cell; iPSC: induced pluripotent stem cell; KC: Kupffer cell; LSEC: liver sinusoidal endothelial cell; NK cell: natural killer Cell; Nrf2: nuclear factor erythroid 2-related factor; PHH: primary human hepatocyte; PPARγ: peroxisome proliferator-activated receptor γ; RP: platelet-rich plasma; TGF- β1: transforming growth factor-beta 1; TIMP-1: tissue inhibitor of metalloproteinase-1.

Human-induced pluripotent stem cells (iPSCs) naturally produce EVs capable of modulating the pro-fibrogenic phenotype of HSCs. Once internalized by HSCs, iPSC-EVs reduced the expression of fibrogenic markers. Genomic analyses and preclinical efficacy studies revealed that iPSC-EVs are rich in miRNAs, preferentially accumulated in the liver following administration, and effectively alleviated liver fibrosis and HSC activation in murine models of liver injury^[88]^. Additionally, exosomes derived from immune cells, such as natural killer (NK) cells, exhibited comparable anti-fibrotic properties. Both *in vitro* and *in vivo* studies demonstrated that NK cell-derived exosomes suppressed TGF-β1-induced HSC proliferation, reduced fibrosis in CCl_4_-treated mice, and inhibited HSC activation^[89]^.

EVs from the body fluids of healthy individuals have demonstrated therapeutic potential in liver fibrosis. Serum-derived EVs, for example, attenuated HSC activation, reduced hepatocyte injury, and mitigated inflammation. These effects are largely attributed to specific miRNAs present in serum EVs, such as miR-34c-3p, miR-151-3p, miR-483-5p, miR-532-5p, and miR-687, which are suppressed in fibrotic conditions^[90]^. Platelet-rich plasma-derived EVs (PRP-EVs) administration in a CCl_4_-induced fibrosis model increased the expression of albumin and vascular endothelial growth factor (VEGF), leading to hepatocyte proliferation. Furthermore, PRP-EVs downregulated TGF-β1 levels and polarized hepatic macrophages to anti-inflammatory M2 phenotype^[91]^. Similarly, milk-derived EVs inhibit HSC activation by downregulating collagen I, while increasing PPARγ expression^[92]^.

### EVs derived from non-animal sources

Recent studies have explored the anti-fibrotic potential of EVs derived from non-animal sources, such as plants and bacteria, in treating liver fibrosis [Table 2]. Plant-derived EVs (PDEVs) contain bioactive molecules like miRNAs, proteins, and lipids that reduce inflammation and modulate oxidative stress pathways, central to liver fibrosis progression^[98,99]^. For instance, EVs derived from tea leaves effectively inhibited HSC activation and attenuated liver fibrosis in a CCl_4_-induced fibrosis model. Moreover, tea leaves-derived EVs delivered miR-44, a molecule shown to suppress fibrogenic pathways^[93]^. Furthermore, cannabis bud-derived EVs have proved hepatoprotective in a non-alcoholic fatty liver disease (NAFLD) model by inhibiting oxidative stress and reducing fibrosis-associated markers^[94]^. Notably, PDEVs exhibit high biocompatibility and low immunogenicity, making them attractive candidates for clinical applications^[99]^. EVs from probiotics, including *Lactobacillus* and *Bifidobacterium* species, further demonstrate therapeutic potential by modulating gut-liver axis communication^[100]^. For instance, EVs derived from *Akkermansia muciniphila*, a gut microbiota member, have been shown to regulate inflammation and promote the regression of activated HSCs. In a high-fat diet and CCl_4_-induced liver injury model, these EVs reduced fibrosis and inflammation biomarkers, ameliorated liver and colon histopathological damage, improved liver function, and normalized inflammatory cytokine levels^[95]^. These findings suggest bacterial EVs as a promising strategy for addressing liver fibrosis through gut-liver axis modulation.

## ENHANCING EV THERAPEUTICS THROUGH CELLULAR PRECONDITIONING AND GENETIC MODIFICATION

**Figure 3:**
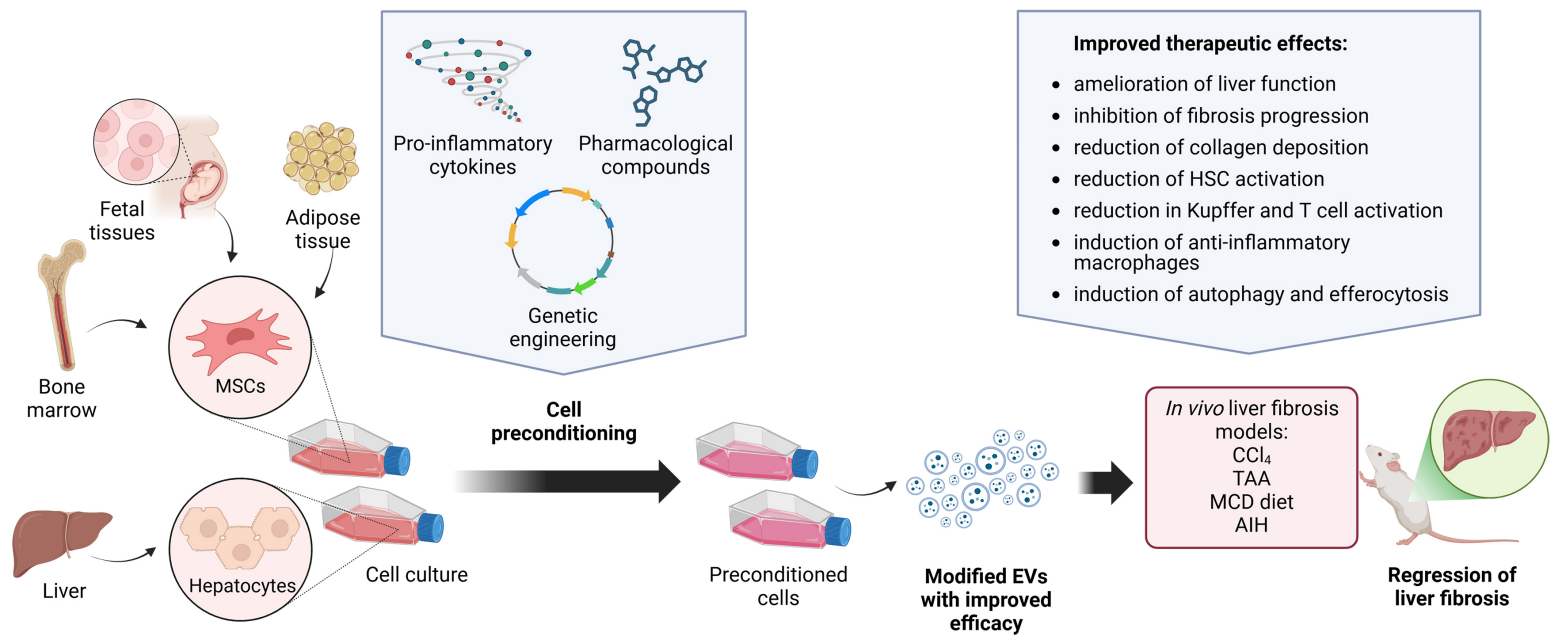
Enhanced effects of EVs derived from preconditioned cells in liver fibrosis. EVs derived from MSCs and hepatocytes can be preconditioned using various strategies, including exposure to pro-inflammatory cytokines (e.g., IFN-γ and TGFβ1), pharmacological compounds, or genetic engineering with lentiviral and adenoviral vectors, to enhance their therapeutic potential. Compared to native EVs, these modified EVs exhibit improved anti-fibrotic and anti-inflammatory effects. The therapeutic efficacy of these preconditioned EVs has been evaluated in in vivo models of liver fibrosis, including CCl_4_, TAA, MCD diet, and AIH. Created using BioRender. Created in BioRender. Chiabotto, G. (2025) https://BioRender.com/7120wd6. AIH: Autoimmune hepatitis; CCl_4_: carbon tetrachloride; EVs: extracellular vesicles; HSC: hepatic stellate cell; MCD: methionine- and choline-deficient; MSCs: mesenchymal stromal cells; TAA: thioacetamide.

Preconditioning of MSCs has emerged as a promising approach to boosting their therapeutic potential in addressing fibrosis and inflammation. Various approaches have been explored to optimize this effect. Hypoxic preconditioning upregulates pro-survival and anti-inflammatory factors^[101,102]^, while exposure to pro-inflammatory cytokines, like TNF-α and IFN-γ, enhances the production of immunomodulatory molecules^[103-106]^. Pharmacological preconditioning using small molecules^[107-109]^ and genetic modification techniques, such as overexpression of specific proteins or miRNAs, have been employed to augment the therapeutic efficacy of MSCs^[110]^. These methods collectively aim to modulate the cargo and functional properties of MSC-EVs, ultimately improving their therapeutic efficacy in treating fibrotic and inflammatory conditions^[111]^ [Table 3 and Figure 3].

**Table 3:** Effect of EVs derived from preconditioned and engineered cells in liver fibrosis

**Model**	**EV name**	**EV sources**	**Preconditioning method**	**Effects of EV-administration**	**Targeted cells**	**References**
CCl_4_	γ-sEVs	ADSC	Preconditioning with IFN-γ	· Induction of anti-inflammatory macrophages · Improvement of inflammation · Reduction of fibrosis	Macrophages Regulatory T cells	[112]
CCl_4_/TAA	WJ-MSCs exosomes	WJ-MSC	Preconditioning with TGFβ1	· Decrease of phospho-Smad2/3 levels · Downregulation of α-SMA, and collagen1 · Upregulation of E-cadherin gene expression	HSCs	[113]
MCD	MSCs/Exo-Cur	CB-MSC	Preconditioning with curcumin	· Amelioration of fibrosis, steatosis and inflammation · Decrease of the serum level of liver enzymes, triglycerides, and cholesterol · Increase of lipid peroxidation · Downregulation of the ASK-JNK-BAX genes	HepG2 cells and liver cells *in vivo*	[114]
CCl_4_	Que-EVs	BMSCs	Preconditioning with quercetin	· Inhibition of M1 macrophage polarization · Reduction of liver fibrosis and inflammation by suppressing the GNAS/PI3K/ERK/STAT3 signaling pathway	Macrophages	[115]
CCl_4_	Exo- miR146 -5p	Hepatocytes	Preconditioning with salidroside	· Inhibition of HSCs activation · Suppression of liver fibrosis · Reduction of EMT process · Decrease of fibrosis-related genes	HSCs	[116]
S100 LPS/ATP	BMSCs-exo^miR-223^	BMSCs	Transfection with lentivirus to overexpress miR-223 or with a miR-223 inhibitor	· Downregulation of inflammatory cytokines · Reduction of liver injury markers · Downregulation of the expression of NLRP3 and caspase-1	Hepatocytes (AML12)	[117]
MCD	HExos	BMSCs	Adenoviral transfection to overexpress HO-1	· Attenuation of IRI · Reduction of ferroptosis · Improvement of liver function · Reduction of activation of Kupffer and T cells · Reduction of long-term biliary fibrosis · Negative regulation of ferroptosis	Hepatocytes Bile duct epithelial cells ACSL4 (Enzyme)	[118]
TAA	miR-148a-5p-Exo	BMSCs	Lentiviral transfection to overexpress miR-148a-5p	· Downregulation of mRNA and protein levels of TGF-β1, TIMP-1, collagen I, and α-SMA	HSCs	[119]
Human HSCs (LX-2)	MSC-circDIDO1 Exo	BMSCs	Transfection with lipofectamine with plasmid encoding circDIDO1	· Suppression of HSC activation through the miR-141-3p/PTEN/AKT pathway	HSCs	[120]
CCl_4_	miR-181-5p-ADSCs Exo	ADSCs	Transfection with lipofectamine to overexpress miR-181-5p	· Downregulation of Stat3 and Bcl-2 · Activation of autophagy · Downregulation of collagen I, vimentin, a-SMA, and fibronectin	HSCs	[121]
CCl_4_	ADSC-122 Exo	ADSCs	Lentiviral transfection of pre-miR-122	· Reduction of the gene expression levels of IGF-1R, CCNG1, and P4HA1 · Reduction of collagen deposition	HSCs	[122]
CCl_4_	ADSC^HGF^-Exo	ADSCs	Lentiviral transfection to overexpress HGF	· Improvement of liver function · Amelioration of histopathological changes in the liver · Upregulation of the expressions of ALB, CK-18, and HNF4α · Downregulation of the expression of α-SMA, collagen I and Rho GTPase	Hepatocytes	[123]
CCl_4_ TGF-β1-activated mouse HSCs (C2211)	ADSC^HGF^-Exo	ADSCs	Lentiviral transfection to overexpress HGF	· Alleviate the activation of HSC · Reduction of the expression of RhoA, Cdc42, and Rac1 · Reduction of the production of collagen	HSCs	[124]
TAA	IGF-1-HUCPVC-EVs	ADSC iMSC HUCPVC	Adenoviral transfection to express IGF-1	· Reduction of collagen I and α-SMA	HSCs	[125]
TAA	IGF-1-HUCPVCs EVs	HUCPVC	Adenoviral transfection to express IGF-1	· Reduction of collagen deposition and expression of pro-fibrogenic genes · Macrophage polarization toward anti-inflammatory phagocytes	HSCs Hepatic macrophages	[126]
CCl_4_	BMP7-EVs	UC-MSCs	Lentiviral transfection to express BMP7	· Modulation of fibrosis cascade response · Reversion of activated HSC phenotype · Suppression of HSC proliferation	HSCs	[127]
CCl_4_	MSC-EV^miR-4465^	UC-MSCs	Transfection with Lipofectamine to overexpress miR-4465	· Reduction of LOXL2 expression · Decrease of HSC activation and collagen production	HSCs	[128]

ACSL4: Acyl-CoA synthetase long chain long chain family member 4; ADSCs: adipose-derived mesenchymal stromal cells; α-SMA: alpha-smooth muscle actin; AKT: protein kinase B; ALB: albumin; AML12: alpha mouse liver 12; ADSC-122: MiR-122-modified ADSCs; ATP: adenosine triphosphate; Bcl2: B-cell lymphoma 2; BMSCs: bone marrow-derived mesenchymal stromal cells; CB-MSC: cord blood mesenchymal stromal cell; CCL4: carbon tetrachloride; CCNG1: Cyclin G1; Cdc42: cell division control protein 42 homolog; Exo: exosome; GPX4: glutathione peroxidase 4; HExos: exosomes derived from heme oxygenase 1 upregulated cells; HGF: hepatocyte growth factor; HNF4α: hepatocyte nuclear factor 4α; HO-1: heme oxygenase 1; HSCs: hepatic stellate cells; IGF-1: insulin-like growth factor 1; UC-MSC: human umbilical cord mesenchymal stromal cell; HUCPVC: human umbilical cord perivascular cell; iMSC: induced-pluripotent-stem-cell-derived mesenchymal stem cell; IRI: ischemia-reperfusion injury; LPS: lipopolysaccharide; MCD: methionine and choline deficient; NLRP3: nucleotide-binding domain, leucine-rich-containing family, pyrin domain-containing-3; PTEN: phosphatase and tensin; P4HA1: prolyl-4-hydroxylase α1; Smad: suppressor of mothers against decapentaplegic; STAT3: signal transducer and activator of transcription 3; TAA: thioacetamide; TGF-β1: transforming growth factor-beta 1; Timp-1: tissue inhibitor of metalloproteinase-1; WJ-MSC: Wharton’s jelly-derived mesenchymal stromal cell.

### Cell preconditioning with pro-inflammatory cytokines

Takeuchi *et al*. demonstrated that EVs from IFN-γ-preconditioned human ADSCs exhibited enhanced therapeutic effects^[112]^. Administering these (γ-EVs) in a murine CCl_4_-induced liver fibrosis model resulted in improved liver function and a marked reduction in fibrosis, with decreased collagen deposition and downregulation of fibrosis-related genes. Additionally, γ-EVs promoted a shift in the liver macrophage population toward an anti-inflammatory phenotype and the expansion of regulatory T cells (Tregs), thus contributing to immune tolerance and fibrosis suppression. At the molecular level, γ-EVs were found enriched with anti-fibrotic miRNAs and proteins. Furthermore, the γ-EVs enhanced efferocytosis, the process by which macrophages clear apoptotic cells, aiding in the resolution of inflammation and preventing chronic fibrosis.

Similarly, Salehipour Bavarsad *et al*. investigated exosomes derived from WJ-MSCs preconditioned with TGFβ1^[113]^. The preconditioning of WJ-MSCs with TGFβ1 altered the exosomal cargo by enriching it with proteins and miRNAs associated with the regulation of fibrogenic pathways in the liver, thus enhancing their therapeutic efficacy. In CCl_4_- and TAA-induced liver fibrosis models, the administration of TGFβ1-preconditioned exosomes significantly reduced liver fibrosis. This anti-fibrotic effect was particularly evident in HSCs, where exosomes downregulated the TGFβ/Smad signaling pathway and promoted the breakdown of ECM components, thereby mitigating fibrosis.

### Cell preconditioning using pharmacological compounds

Tawfeek *et al*. explored exosomes derived from human cord blood mesenchymal stromal cells (CB-MSCs) preconditioned with curcumin (MSCs/Exo-Cur) for treating MCD-induced liver fibrosis^[114]^. Administration of MSCs/Exo-Cur significantly reduced hepatic lipid accumulation, improved liver function, and exhibited prolonged anti-fibrotic effects. Unlike unconditioned MSC-derived exosomes, which provided only temporary benefits, MSCs/Exo-Cur offered sustained protection against fibrosis both *in vitro* and *in vivo*, suggesting that curcumin preconditioning significantly enhances their anti-inflammatory, anti-oxidant, and anti-fibrotic properties. Besides curcumin, another well-known compound with broad pharmacological activities is quercetin (Que). Jiang *et al*. demonstrated that priming BMSCs with this flavonoid enhances the anti-fibrotic effects of their EVs^[115]^. Notably, Que preconditioning increased levels of the anti-inflammatory miR-136-5p in BMSC-EVs. In a CCl_4_-induced liver fibrosis model, Que-BMSC-EVs inhibited M1-type macrophage polarization and alleviated liver inflammation by suppressing the GNAS/PI3K/ERK/STAT3 signaling pathway. Another study by Lang *et al*. examined the efficacy of exosomes derived from hepatocytes preconditioned with Salidroside, a natural anti-inflammatory and anti-oxidant compound, in liver fibrosis^[116]^. Preconditioning of hepatocytes with Salidroside enhanced the bioactive properties of their exosomes, making them more effective in mediating the crosstalk between hepatocytes and HSCs. Upon administration in a CCl_4_-induced liver fibrosis model, exosomes derived from Salidroside-preconditioned hepatocytes effectively inhibited the activation of HSCs and suppressed the EMT, a crucial step in fibrosis progression, and downregulated the expression of fibrosis-related genes in HSCs. By delivering anti-fibrotic miRNAs such as miR-146a-5p, these exosomes could modulate HSC behavior, thus reducing fibrogenic activity and mitigating the fibrotic response in the liver.

### Cell preconditioning through genetic engineering

Another promising preconditioning strategy involves the use of genetically engineered MSCs as an EV source. Chen *et al*. generated a murine model featuring major pathologic characteristics of AIH through the injection of hepatic S100^[117]^. Then, they tested the *in vivo* efficacy of exosomes derived from BMSC transfected with a lentiviral vector to overexpress miR-223. These miR-223-enriched exosomes ameliorated liver function and reduced inflammation, as evidenced by decreased levels of pro-inflammatory cytokines and essential inflammatory mediators, including NLRP3 and caspase-1, involved in the inflammasome pathway. This dual action of reducing inflammation and modulating immune responses highlights the role of miR-223-enriched exosomes in mitigating AIH progression. The downregulation of NLRP3 and caspase-1 induced by miR-223-enriched exosomes was also validated *in vitro* using the murine hepatocyte cell line AML12.

Fatty liver disease presents significant challenges in transplantation, mainly due to the increased susceptibility to ischemia-reperfusion injury (IRI). This heightened susceptibility complicates the post-transplant recovery process, contributing to poorer outcomes. To address this issue, Tian *et al*. explored the therapeutic potential of exosomes isolated from BMSCs genetically modified through adenoviral transfection to overexpress heme oxygenase 1 (HO-1), an enzyme known for its cytoprotective and anti-inflammatory properties^[118]^. In a rat model of hepatic steatosis induced by a MCD diet, which closely mimics the clinical scenario of fatty liver transplantation, HO-1-overexpressing exosomes prevented the development of biliary fibrosis, improved liver function, and attenuated IRI by reducing oxidative stress and ferroptosis, as evidenced by decreased levels of markers of oxidative damage, such as malondialdehyde (MDA) and iron. Furthermore, EV treatment also led to decreased activation of Kupffer cells and reduced infiltration of T cells, suggesting a dampened inflammatory response.

In another study, Xuan *et al*. used lentiviral transfection to generate miR-148a-5p-overexpressing BMSCs, demonstrating significant anti-fibrotic effects in a TAA-induced liver fibrosis model^[119]^. Exosomes from engineered BMSCs improved overall liver function and attenuated fibrosis by delivering miR-148a-5p directly into HSCs and downregulating Smad4, a key signaling molecule in the TGF-β1 pathway. This led to a decrease in pro-fibrotic markers expression, thereby reducing ECM deposition and ameliorating fibrosis.

Beyond miRNAs, circular RNAs (circRNAs) also play a crucial role in EV-mediated anti-fibrotic effects. Ma *et al*. demonstrated that exosome-mediated delivery of circDIDO1 effectively inhibited HSC activation^[120]^. This specific circRNA, which is downregulated in the serum of liver failure patients, acts as a sponge for miR-141-3p, elevating PTEN levels and suppressing the AKT signaling pathway, ultimately reducing the expression of pro-fibrotic markers like α-SMA and collagen I in HSCs.

Qu *et al*. investigated the therapeutic potential of exosomes derived from miR-181-5p-modified ADSCs in liver fibrosis^[121]^. In a CCl_4_-induced liver fibrosis model, these exosomes attenuated liver injury and significantly downregulated fibrotic markers. *In vitro*, miR-181-5p-enriched exosomes suppressed HSC activation and significantly enhanced autophagy by inhibiting the STAT3/Bcl-2/Beclin 1-dependent pathway. Similarly, Lou *et al*. demonstrated that lentiviral transfection of ADSCs with miR-122 improved the therapeutic potential of their exosomes^[122]^. In a CCl_4_-induced liver fibrosis model, miR-122-enriched exosomes suppressed HSC activation and reduced collagen deposition. *In vitro*, these exosomes enhanced the G0/G1 arrest of HSCs by regulating key miR-122 target genes, such as insulin-like growth factor receptor 1 (*IGF-1R*), Cyclin G1 (*CCNG1*), and prolyl-4-hydroxylase α1 (*P4HA1*), which are involved in HSC proliferation and collagen maturation. Furthermore, exosomes derived from ADSCs transfected with a lentiviral vector to overexpress hepatocyte growth factor (HGF) exhibited enhanced anti-fibrotic properties^[123,124]^. Compared to exosomes from unmodified ADSCs, those from HGF-overexpressing ADSCs (ADSC^HGF^-Exo) demonstrated superior efficacy in reducing the accumulation of collagen and restoring liver function. Besides the reduction of hepatic fibrosis-related proteins, ADSC^HGF^-Exo treatment also downregulated Rho GTPase proteins, specifically CDC42 and Rac1, both involved in the progression of liver injury^[123]^. *In vitro*, ADSC^HGF^-Exo effectively attenuated cell viability, induced cell cycle arrest at the S phase, and promoted apoptosis of TGF-β1-activated HSCs^[124]^. Compared to those from unmodified ADSCs, ADSC^HGF^-Exo showed a stronger suppressive effect on the expression of crucial fibrogenic markers in TGF-β1-treated HSCs.

The engineering of human umbilical cord perivascular cells (HUCPVCs) with adenoviruses coding for insulin-like growth factor 1 (IGF-1) produced IGF-1-enriched EVs with enhanced anti-fibrotic effects. In a TAA model, treatment with IGF-1-enriched EVs reduced fibrosis and promoted liver regeneration. *In vitro*, these engineered EVs reduced HSC activation, as shown by decreased expression of fibrosis-related markers^[125,126]^. Besides HSCs, IGF-1-enriched EVs also targeted hepatic macrophages^[126]^. Moreover, Zhu *et al*. employed lentiviral transfection to produce human UC-MSCs overexpressing the anti-fibrotic bone morphogenetic protein 7 (BMP7)^[127]^. Compared to EVs from unmodified UC-MSCs, BMP7-enriched EVs showed superior targeting capabilities and anti-fibrotic effects in a CCl_4_ model. These EVs predominantly accumulated in fibrotic liver tissue. *In vitro*, BMP7-loaded EVs promoted the phenotypic reversal of activated HSCs and inhibited their proliferation. Wang *et al*. engineered UC-MSCs to secrete miR-4465-enriched EVs, aiming to inhibit liver fibrosis by targeting lysyl oxidase-like protein 2 (LOXL2) expression^[128]^. *In vitro*, miR-4465-modified MSC-EVs (MSC-EV^miR-4465^) effectively delivered miR-4465 into HSCs, leading to reduced LOXL2 expression, decreased HSC activation, and lower collagen production. In a CCl_4_-induced liver fibrosis mouse model, treatment with MSC-EV^miR-4465^ resulted in diminished HSC activation and collagen deposition.

## EVs AS DRUG-DELIVERY SYSTEMS IN LIVER FIBROSIS

**Figure 4:**
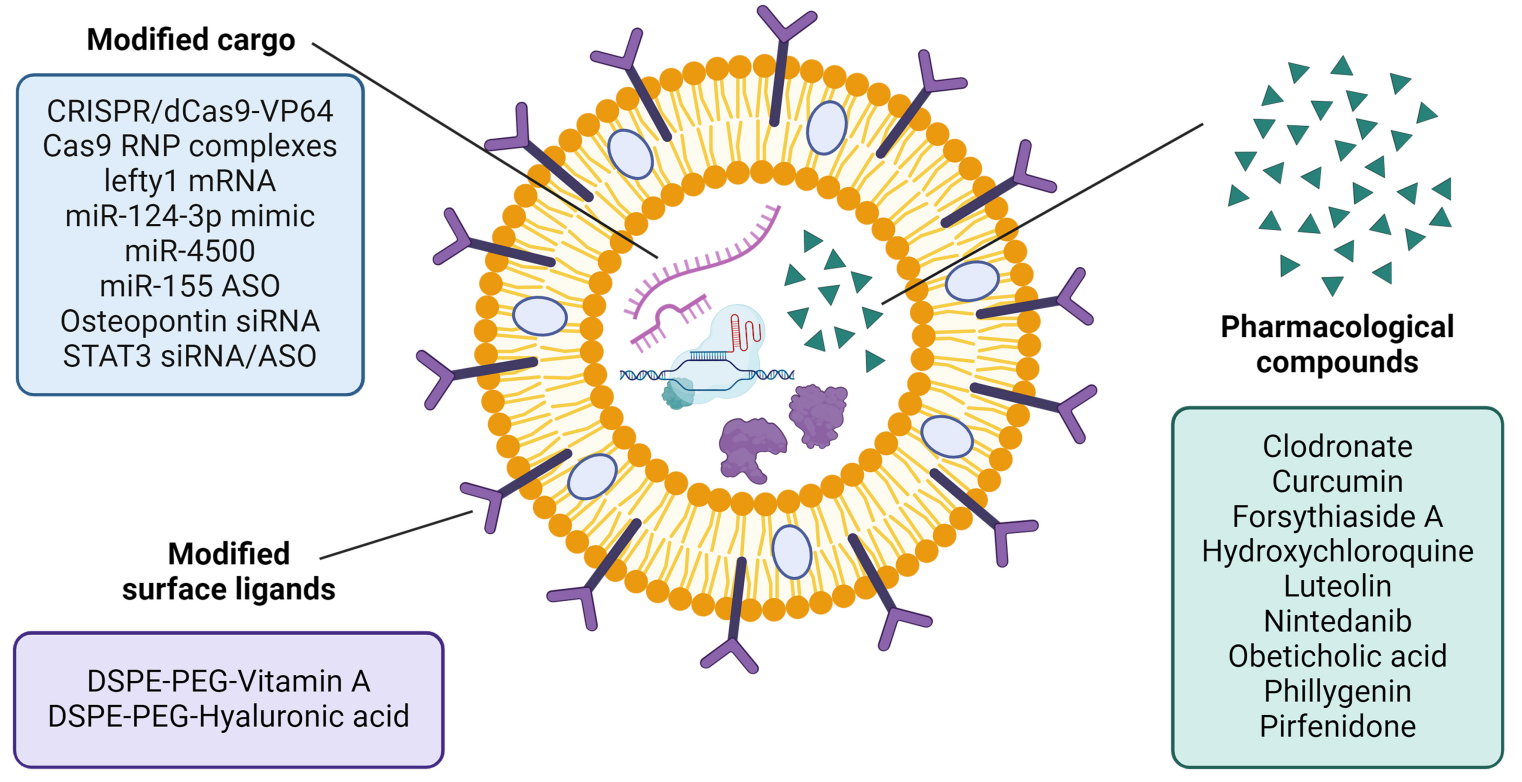
EV modifications to enhance drug delivery in liver fibrosis. Different engineering strategies used to optimize EVs for drug delivery in liver fibrosis. EVs can be modified to improve their targeting ability and enhance cargo loading. Various molecular cargoes, including small molecules, RNAs, and proteins, can be loaded into EVs to counteract liver fibrosis. The combination of these modifications enables the development of more efficient and precise EV-based drug delivery systems for liver fibrosis treatment. Created using BioRender. Created in BioRender. Chiabotto, G. (2025) https://BioRender.com/nblg6av. ASO: Antisense oligonucleotide; Cas9: CRISPR-associated protein 9; CRISPR: clustered regularly interspaced short palindromic repeats; DSPE-PEG: 1,2-distearoyl-sn-glycero-3-phosphoethanolamine-polyethylene glycol; EV: extracellular vesicle; mRNA: messenger RNA; siRNA: small interfering RNA; STAT3: signal transducer and activator of transcription 3; VP64: viral protein 64.

EVs represent a new generation of targeted drug delivery carriers that can contribute to the improvement of therapeutic strategies for human diseases, such as liver fibrosis^[129]^. Considering their biocompatibility, capacity to cross the blood-brain barrier, intrinsic targeting ability, and involvement in cell-to-cell communications, EVs show different advantages over artificial systems^[130]^. The application of EVs loaded with drugs, RNA interference molecules, miRNAs, and proteins represents a promising frontier in treating acute and chronic liver diseases^[131]^ [Figure 4 and Table 4]. This innovative potential of modified EVs in drug delivery and regenerative medicine opens a world of possibilities, inspiring further research and development.

**Table 4:** EVs as drug delivery systems in liver fibrosis

**Model**	**Loading protocol**	**EV sources/Isolate**	**Loaded components**	**Effects of EV-administration**	**Targeted cells**	**References**
**MSC-EVs**						
Murine CCl_4_	Sonication	Human WJ-MSCs	OCA	· Activation of the FXR-Cyp7a1 cascade to reduce liver fibrosis · Prevention of HSC activation · ECM remodelling	HSCs	[132]
Murine CCl_4_	CaCl_2_	Human WJ-MSCs	miR-124-3p mimic	· Downregulation of STAT3 signaling · Downregulation of pro-inflammatory and pro-fibrotic markers	HSCs Ly6C^hi^ monocytes	[133]
Murine CCl_4_	Electroporation	Human BMSCs	siRNA or ASO targeting STAT3	· Downregulation of STAT3 · Amelioration of liver function and morphology · Inhibition of HSC activation	HSCs	[134]
Rat CCl_4_	Sonication and physical incubation	Rat BMSCs	LUT	· Amelioration of liver function · Reduction of collagen deposition · Reduction of immune cell infiltration · Downregulation of TNF-α, MMP-2 and TGF-β	HepG2	[135]
Murine CCl_4_	Electroporation	Human ADSCs	siRNA targeting OPN	· Inhibition of HSC proliferation · Reduction of collagen deposition and α-SMA expression · Inhibition of TGF-β1 signaling	HSCs	[136]
Murine TAA	Phospholipid insertion method	Human ADSCs	DSPE-PEG-Vitamin A on the surface	· Inhibition of HSC activation · Downregulation of α-SMA, p-SMAD2, TIMP-1 and LYC6 protein expression · Upregulation of CK18 protein expression	HSCs	[137]
**EVs from other cell sources**						
Rat TAA	Electroporation	Rat primary hepatocytes	miR-4500	· Targeting of TGF-βR1 · Inhibition of HSC activation · Attenuation of liver fibrosis, oxidative stress and angiogenesis	HSCs	[138]
Murine CCl_4_	CRISPR/dCas9 technology	Murine hepatocytes (AML12)	CRISPR/dCas9-VP64	· Activation of HNF4α · Downregulation of α-SMA and collagen I protein expression	HSCs	[139]
Murine APAP-ALI, CCl_4_, orthotopic model	Electroporation	Human HSCs (LX-2)	Cas9 RNP complexes	· Downregulation of PUMA, Cyclin E1 and KAT5 · Inhibition of HSC activation · Amelioration of liver fibrosis	Hepatocytes and HSCs	[140]
Rat CCl_4_	Electroporation	Rat HSCs (HSC-T6)	lefty1 mRNA	· Inhibition of HSC activation · Downregulation of α-SMA, collagen I, and TIMP-1 and MMP-1 · Inhibition of TGF-β/SMAD signaling	HSCs	[141]
Rat CCl_4_	Co-incubation and ultrasound cycles	Rat HSCs (HSC-T6)	PFD; DSPE-PEG2000-HA on the surface	· Inhibition of HSC activation · Amelioration of hepatic cell morphology · Amelioration of liver fibrosis	HSCs	[142]
Zebrafish TAA	EDC/NHS chemistry; co-incubation in ultrasonic conditions	Milk	FA; DSPE-PEG2000-HA on the surface	· Inhibition of TGF-β1-induced HSC proliferation · Downregulation of α-SMA and collagen protein · Amelioration of liver function and morphology	HSCs	[143]
Zebrafish TAA	EDC/NHS chemistry; co-incubation	Milk	PHI; DSPE-PEG2000-HA on the surface	· Inhibition of HSC activation · Downregulation of autophagy · Amelioration of liver function	HSCs	[144]
Murine CCl_4_	Co-incubation and saponin-mediated loading	Milk	Curcumin	· Reduction of serum markers of liver function · Reduction of ECM components	Hepatocytes	[145]
Murine ALF and orthotopic model	Electroporation	RBC	miR-155-ASO, doxorubicin, sorafenib	· Amelioration of liver function and histology · Inhibition of tumor growth and angiogenesis · Induction of polarization of M2 macrophages	M1 macrophages, tumoral cells	[146]

ALI: Acute liver injury; ALF: acute liver failure; ADSCs: adipose-derived mesenchymal stromal cells; AML12: alpha mouse liver 12; APAP: acetaminophen; α-SMA: alpha-smooth muscle actin; ASO: antisense oligonucleotide; BMSCs: bone marrow-derived mesenchymal stromal cells; Casp9: caspase 9; CCl4: carbon tetrachloride; CK18: cytokeratin 18; CRISPR: clustered regularly interspaced short palindromic repeats; dCasp9: dead caspase 9; DSPE-PEG: 1, 2-distearoyl-sn-glycero-3-phosphoethanolamine-poly(ethylene glycol); ECM: extracellular matrix; EDC: 1-ethyl-3-(3-dimethylaminopropyl) carbodiimide; FA: forsythiaside A; FXR-Cyp7a1: farnesoid X receptor-cytochrome P450 7A1; HA: hyaluronic acid; HepG2: human hepatoma cell line; HNF4α: hepatocyte nuclear factor 4α; HSC-T6: rat hepatic stellate cells; HSTP1: hematopoietic stem cell thrombopoietin receptor 1; KAT5: lysine acetyltransferase 5; LYC6: lymphocyte antigen 6 family member C1; LUT: luteolin; miR: microRNA; MMP: matrix metalloproteinase; MSC: mesenchymal stromal cells; NHS: N-hydroxy succinimide; OCA: obeticholic acid; OPN: osteopontin; PFD: pirfenidone; PHI: phillygenin; p-SMAD 2: phosphorylated-suppressor of mothers against decapentaplegic 2; PUMA: p53 upregulated modulator of apoptosis; RBC: red blood cell; RNP: ribonucleo protein; SMAD: suppressor of mothers against decapentaplegic; siRNA: small interfering RNA; STAT3: signal transducer and activator of transcription 3; TAA: thioacetamide; TGF-β1: transforming growth factor-beta 1; TGF- βR1: transforming growth factor-beta receptor 1; TIMP-1: tissue inhibitor of metalloproteinase-1; TNF-α: tumor necrosis factor alpha; WJ-MSC: Wharton’s jelly-derived mesenchymal stromal cell.

### Modified MSC-EV applications in liver fibrosis

MSC-EVs can be modified to load small-molecule payloads, enhancing their anti-fibrotic properties^[147]^. Their small size, straightforward structure, and low immunogenicity further underscore their strong abilities as regulatory factors and transporters of signaling molecules^[148]^. As reported in Table 4, different studies delve into groundbreaking therapeutic approaches for liver fibrosis using EVs derived from MSCs as delivery systems for different therapeutic molecules. EVs derived from h-WJ-MSCs loaded with obeticholic acid (OCA), a farnesoid X receptor (FXR) agonist with hepatoprotective properties, were administered in a CCl_4_-induced liver fibrosis murine model^[132]^. Exo-loaded OCA accumulated in the liver and effectively improved liver function and mitigated tissue fibrosis compared to free OCA. Through the activation of FXR-Cyp7a1 cascade, Exo-loaded OCA was able to prevent HSC activation and modulate the expression of genes and proteins involved in ECM remodeling, facilitating hepatic recovery.

Considering that the aberrant expression of miR-124-3p is associated with the progression of liver diseases, Niknam *et al*. produced miR-124-3p-enriched exosomes (ExomiR-124), which were administered to CCl_4_-mice for three weeks^[133]^. ExomiR-124 improved hepatic fibrosis by reducing the production of pro-inflammatory cytokines and pro-fibrotic markers, particularly collagen. Furthermore, ExomiR-124 induced the splenic monocytes to acquire a restorative Ly6Clo phenotype and downregulated Signal transducer and activator of transcription 3 (STAT3) levels, which contributed to the inhibition of α-SMA gene expression, resulting in HSC deactivation. As mentioned above, STAT3 is a key regulator of liver fibrosis; in fact, it is involved in the activation of fibroblasts and HSCs, which acquire a myofibroblast-like phenotype^[149,150]^. In the study conducted by Tang *et al*., EVs were purified from human-BM-MSCs, and they were electroporated with small interfering RNAs (siRNA) (iExo^siRNA-STAT3^) or antisense oligonucleotides specifically targeting STAT3 (iExo^mASO-STAT3^)^[134]^. *In vitro* experiments demonstrated that iExo^siRNA-STAT3^ and iExo^mASO-STAT3^ effectively targeted activated HSCs and reduced the mRNA levels of STAT3. *In vivo*, an amelioration of liver function and morphology was observed in the CCl-4 murine model, mainly due to the reduction in ECM deposition. Transcriptomic analyses on liver tissue revealed that modified EVs improved liver function by downregulating genes involved in fibrosis-associated pathways, including ECM accumulation, HSC activation, and inflammation. These results suggested that the EV-mediated delivery of STAT3-targeting therapeutics could represent a highly innovative and promising strategy for treating chronic fibrotic liver diseases.

In another research study, EVs were isolated from rat-BMSCs and loaded with luteolin (LUT), a flavonoid found in many edible plants, which possesses anti-inflammatory and anti-oxidant properties, and is known to alleviate liver fibrosis by inhibiting the proliferation of activated HSCs^[151,152]^. In a rat CCl_4_-induced model of liver fibrosis, Ashour *et al*. demonstrated that luteolin-loaded exosomes (LUT-Ex) reduced the expression of pro-inflammatory marker TNF-α and pro-fibrotic markers hydroxyproline, MMP-2, and TGF-β^[135]^. Compared to LUT-suspension and to blank EVs, LUT-Ex induced a stronger improvement of hepatic function, and a greater anti-fibrotic activity, as shown by the decreased immune cell infiltration and collagen deposition in fibrotic livers.

Other research studies investigate EVs isolated from ADSCs as a delivery platform for siRNA-based therapy targeting key regulatory genes in liver fibrosis. For example, high plasma levels of osteopontin (OPN) correlate with advanced fibrosis progression^[153]^, and a similar trend was also observed in patients with NAFLD^[154]^ and chronic HBV-induced fibrosis^[136]^. Tang *et al*. demonstrated that exosome-mediated delivery of a siRNA targeting OPN (iExo^siRNA-OPN^) inhibited ECM deposition in a CCl_4_-induced liver fibrosis model^[136]^. *In vitro* results demonstrated how iExo^siRNA-OPN^ were taken up by HSCs and reduced their proliferative capacity. Interestingly, by speculating the mechanism of action of iExo^siRNA-OPN^, Tang *et al*. highlighted the inhibition of TGF-β1 expression due to the blocking of HMGB1 translocation to the cytoplasm, resulting in HSC inactivation and reduced liver fibrosis. Interestingly, EVs isolated from ADSCs and coupled with vitamin A (V-EVs) showed potent anti-fibrotic effects in liver fibrosis^[136]^. You *et al*. demonstrated that V-EVs were selectively taken up by activated HSCs, thus reducing the deposition of ECM and inhibiting the activation and proliferation of HSCs in a TAA-induced liver fibrosis model^[137]^. Furthermore, the administration of V-EVs resulted in a decrease in the number of LY6Chi macrophages, responsible for the phagocytosis of damaged hepatocytes, and consequently in the production of pro-inflammatory cytokines, thus contributing to hepatocyte regeneration.

### Modified EVs from other sources in liver fibrosis

In addition to MSC-EVs, EVs from other cell sources have also been modified to enhance drug delivery for liver fibrosis treatment. Yang *et al*. developed a ROS-responsive injectable hydrogel encapsulating hepatocyte-derived exosomes loaded with miR-4500 (QCG@Exos^miR-4500^)^[138]^. This hydrogel, formed through dynamic crosslinking of quaternized chitosan and carboxyphenylboronic acid-modified gelatin, specifically targets activated HSCs, delivering miR-4500 to inhibit their activation and promote apoptosis. In a TAA-induced liver fibrosis model, this treatment effectively reduced collagen deposition and improved liver function.

Luo *et al*. isolated EVs from murine hepatocytes (AML12), and they were loaded with CRISPR/dCas9-VP64 (clustered regularly interspaced short palindromic repeats/associated protein) system to test a new therapeutic strategy for liver fibrosis^[139]^. Interestingly, this system was efficiently transferred into HSCs both *in vitro* and *in vivo*, inducing an amelioration of liver fibrosis. The researchers observed that the EV-mediated delivery of the CRISPR/dCas9-VP64 system to activated HSCs *in vivo* induced the reduction of the mRNA and protein levels of α-SMA and collagen I, providing a promising gene therapy strategy for treating hepatic fibrosis through HSC reprogramming.

The EV-mediated delivery system for *CRISPR-Cas9* gene editing was also employed on EVs isolated from LX-2. Wan *et al*. successfully loaded CRISPR-Cas9 ribonucleoprotein (RNP) into purified HSC-derived EVs (exosome^RNP^)^[140]^. *In vitro,* exosome^RNP^ efficiently delivered RNP in the cytosol of HSCs and, *in vivo*, they accumulated in the hepatic tissue. This EV-mediated delivery of Cas9 RNP showed significant therapeutic potential in a mouse model of a single overdose of APAP-induced acute liver injury via targeting of p53-upregulated modulators of apoptosis (PUMA). In addition, exosome^RNP^ targeted cyclin E1 (*CcnE1*) and K (lysine) acetyltransferase 5 (*KAT5*) in a murine model of CCl_4_-induced chronic liver injury and an orthotopic murine model of HCC, respectively. Cyclin E1 is a protein kinase that regulates the proliferation of HSCs^[155]^ and its genome editing mediated by exosome^RNP^ resulted in a significant reduction of HSC activation, with consequent attenuation of liver fibrosis^[140]^.

Other studies further explore the therapeutical application of EVs isolated from HSCs. For instance, Zhao *et al*. employed EVs from rat HSC-T6 cells to deliver mRNA of left-right determination factor (lefty) 1, which inhibits collagen synthesis driven by TGF-β1^[141]^. These lefty1-loaded EVs were effectively taken up by HSCs and reduced their activation, as indicated by decreased levels of α-SMA, collagen I, TIMP-1, and MMP-1. Additionally, administration of lefty1-loaded EVs significantly improved liver fibrosis induced by CCl_4_, inhibiting the TGF-β1 signaling pathway. Moreover, Yu *et al*. encapsulated pirfenidone (PFD) within EVs isolated from rat HSC-T6 cells modified with hyaluronic acid (HA) to specifically target activated HSCs^[142]^. *In vitro* studies demonstrated that these modified EVs (HA@EVs-PFD) effectively inhibited HSC activation more efficiently than free PFD. In a CCl_4_-induced hepatic fibrosis model, four weeks of HA@EVs-PFD treatment resulted in reduced liver collagen deposition and improved hepatic cell morphology.

Milk-derived EVs were also explored as drug delivery systems to treat liver diseases. Gong *et al*. described a targeted delivery method based on milk-derived EVs to encapsulate and administer Forsythiaside A (FA), an active herbal ingredient of traditional Chinese medicine *Forsythiae Fructus* with hepatoprotective properties^[143]^. In this study, the researchers developed CD44-specific ligand HA-modified milk-derived exosomes (mExo) loaded with FA (HA-mExo-FA). *In vitro,* HA-mExo-FA inhibited the proliferation of LX-2 and reduced the gene and protein levels of both α-SMA and collagen. *In vivo,* the researchers developed a zebrafish TAA-induced liver fibrosis model and observed the ability of HA-mExo-FA to improve hepatic function and morphology compared to free FA. Interestingly, the same research group conjugated milk-derived EVs with Hyaluronic acid (DSPE-PEG2000-HA) on their surface and loaded them with Phillygenin (PHI), a hepatoprotective agent with low water solubility^[144]^. PHI-HA-mEXO could specifically bind to the surface of activated HSCs and enter through CD44-mediated endocytosis. PHI-HA-mEXO exhibited a beneficial anti-fibrotic effect by partially inhibiting HSC activation through the downregulation of autophagy. Another research study unveils a promising method for delivering natural compounds. To improve curcumin bioavailability and therapeutic efficacy for the treatment of chronic liver disease, Albaladejo-García *et al*. encapsulated curcumin within small milk EVs (sEVCur)^[145]^. In a CCl_4_-induced liver fibrosis model, sEVs loaded with curcumin via active cargo loading with saponin (sEVCurAc) significantly decreased both the serum markers of liver damage and the deposition of ECM components compared to free curcumin.

In the last years, different studies have highlighted the ability of red blood cell-derived EVs (RBC-EVs) as drug delivery carriers due to their ability to accumulate in the liver, as well as their biocompatibility and safety in transporting bioactive molecules, including RNAs^[156]^. Zhang *et al*. observed that RBC-EVs accumulate in the liver through macrophage-dependent mechanisms^[146]^. Considering this aspect, they investigated whether RBC-EVs could serve as natural drug delivery systems for treating liver diseases. To test this hypothesis, RBC-EVs were electroporated with FAM-labeled miR-155-ASOs and their protective effects were evaluated in a murine model of acute liver failure. By regulating gene expression, RBC-EVs/miR-155-ASOs promoted macrophage polarization toward the anti-inflammatory M2 phenotype, ameliorating hepatic function and histology. Moreover, the researchers loaded RBC-EVs with doxorubicin (RBC-EVs/Dox) and sorafenib (RBC-EVs/SRF) to assess their therapeutic efficacy compared to free drugs in an orthotopic liver cancer mouse model. RBC-EVs/Dox inhibited tumoral growth *in vivo* and suppressed HCC-LM3 cell proliferation, while RBC-EVs/SRF reduced angiogenesis both *in vitro* and *in vivo*.

## EV-LIPOSOME HYBRID DELIVERY SYSTEMS IN LIVER FIBROSIS

The development of EV-liposome hybrid delivery systems has unveiled promising therapeutic opportunities for liver fibrosis, combining the unique attributes of EVs and liposomes. These hybrid systems integrate the targeting precision and biocompatibility of EVs with the high drug-loading capacity and stability of liposomes, creating an advanced platform for the precise delivery of anti-fibrotic agents^[157]^. Moreover, their surface can be functionalized with ligands or antibodies to enhance specificity toward fibrotic liver tissue, ensuring precise drug delivery while minimizing systemic toxicity^[158]^. Additionally, EVs naturally exhibit a longer half-life *in vivo* compared to synthetic nanoparticles, preserving the therapeutic efficacy of encapsulated drugs. Their fusion with liposomes further strengthens structural integrity and improves drug retention, ensuring targeted delivery to liver-resident cells^[159]^.

One prominent example is the HCQ@VA-Lip-Exo system developed by Zhang *et al*.^[160]^. This innovative system employs hydroxychloroquine (HCQ) as an autophagy inhibitor to target activated HSCs. By modifying liposomes with vitamin A and fusing them with BMSC-EVs, this dual-membrane platform efficiently delivers HCQ to activated HSCs while minimizing damage to surrounding hepatic cells. Beyond HCQ’s anti-fibrotic effects, the EV component provides synergistic therapeutic benefits, such as reversing HSC activation and suppressing their proliferation and differentiation. Another cutting-edge application involves the delivery of a combined regimen of clodronate and nintedanib via EV-liposome hybrids^[161]^. This approach specifically targets Kupffer cells, mitigating their pro-inflammatory activity and reducing liver fibrosis progression.

EV-liposome hybrids overcome several limitations of standalone EVs or liposomes, including suboptimal bioavailability, rapid clearance, and off-target effects. A key advantage of these hybrid systems is their ability to overcome biological barriers, such as the sinusoidal endothelial capillary barrier in the liver, which often hinders the penetration of therapeutic agents. It has been shown that hybrid vesicles can effectively navigate this barrier, delivering therapeutic molecules directly to activated HSCs and reducing collagen deposition and fibrotic progression^[162]^. Furthermore, functionalization with biomimetic coatings, such as neutrophil membranes, increases their targeting efficiency toward fibrotic regions while aiding immune evasion. However, potential challenges and limitations of these hybrid systems, such as immune responses or unexpected interactions between the EVs and the liposomes, should be carefully evaluated in future research to ensure their safety and efficacy in liver fibrosis therapy.

## CONCLUSION

Numerous studies highlight the potential of EVs from different sources as promising therapeutic tools to treat liver diseases, in particular hepatic fibrosis. Compared to the cells of origin, EVs are stable, biocompatible and act as carriers of biologically active molecules (mRNAs, proteins, and non-coding RNAs), which are transferred to the target cell, influencing its pathophysiological state. In the context of liver fibrosis, the EV mechanisms of action include suppression of the expression of TGF-β and of HSC activation, regulation of programmed cell death, and reduction of inflammation, contributing to hepatic regeneration and preservation of tissue integrity. Among the different sources, MSC-EVs demonstrate significant therapeutic potential, which can be improved using different approaches, including the preconditioning of the cell of origin, to enhance their efficacy. Various preconditioning strategies, such as exposure to pro-inflammatory cytokines, pharmacological treatments, and genetic modifications, have been explored to optimize the therapeutic efficacy of cell-derived EVs.

EVs also represent a promising drug delivery system that is able to improve the pharmacokinetic and pharmacodynamic properties of medications. Loading EVs with different types of molecules, including drugs, RNA interference molecules, and therapeutic miRNAs, offers several advantages, such as deep tissue penetration, sustained cargo release, prolonged circulation time, safety, and biocompatibility. Additionally, EV surface engineering improves their targeting ability, ensuring the precise and efficient delivery of therapeutic cargo to liver cells, in particular HSCs and macrophages, thereby alleviating liver fibrosis. Furthermore, combining natural or engineered EV formulations with existing anti-fibrotic, immunomodulatory, or anti-inflammatory treatments may further enhance their efficacy in ameliorating liver morphology and function. All these approaches could pave the way for highly specific and effective therapies, not only for liver fibrosis but also for other fibrotic diseases characterized by excessive ECM deposition.

Although EVs have demonstrated promising biological activity in several preclinical models of liver disease, further research is needed to address several challenges related to their clinical application. First, there is a need for the standardization and large-scale production of EVs in accordance with current good manufacturing practices (CGMP). Achieving CGMP compliance to produce clinical-grade EVs requires adherence to strict regulatory standards, including the establishment of a qualified cell bank. Among the available isolation methods, differential ultracentrifugation represents the most widely used; however, it is time-consuming and cost-inefficient for large-scale processing and does not align with CGMP regulations. Size-exclusion chromatography, while CGMP-compatible, lacks scalability. In the last years, tangential flow filtration has emerged as an attractive alternative due to its ability to process larger sample volumes for clinical-grade EV production, although an additional ultracentrifugation step is often required to concentrate the final product.

Another critical aspect is optimizing EV delivery to ensure effective targeting of the desired cell populations. Natural EV tropism toward certain organs, like the liver, as well as the administration regimen, can influence therapeutic efficacy. However, the sequestration of EVs within the hepatic parenchyma may reduce bioavailability and limit their interaction with key cellular components required to induce a regenerative response. Engineering techniques, such as functionalizing the EV surface with targeting moieties, could refine cell specificity and increase their therapeutic ability.

Finally, standardized quality control measures are essential to assess product stability. Defining optimal storage conditions, including temperature ranges and the use of cryopreservatives, is crucial for preserving EV bioactivity, and ensuring safety for their translation to the clinic. Furthermore, for future clinical applications, efforts should focus on clarifying EV mechanisms of action, standardizing isolation protocols, and establishing comprehensive CGMP guidelines to facilitate regulatory approval and widespread therapeutic use.

## DECLARATIONS

### Copyright

© The Author(s) 2025.
